# The Pull-Back Technique for the 532 Slim Modiolar Electrode

**DOI:** 10.1155/2019/6917084

**Published:** 2019-05-23

**Authors:** C. Riemann, H. Sudhoff, I. Todt

**Affiliations:** Department of Otolaryngology, Head and Neck Surgery, Klinikum Bielefeld, Ruhr Universität Bochum, Germany

## Abstract

**Introduction:**

The distance between the modiolus and the electrode array is one factor that has become the focus of many discussions and studies. Positioning the electrode array closer to the spiral ganglion with the goal of reducing the current spread has been shown to improve hearing outcomes. The perimodiolar electrode arrays can be complemented with a surgical manoeuvre called the pull-back technique. This study focuses its attention on the recently developed 532 slim modiolar electrode.

**Objective:**

To investigate the intracochlear movements and pull-back technique for the 532 slim modiolar electrode.

**Material and Methods:**

A decapping procedure of the cochlea was performed on 5 temporal bones. The electrode array was inserted, and the intracochlear movements were microscopically examined and digitally captured. Three situations were analysed: the initial insertion, the overinsertion, and the pull-back position. The position of the three white markers of the electrode array in relation to the round window (RW) was evaluated while performing these three actions.

**Results:**

The initial insertion achieved an acceptable perimodiolar position of the electrode array, but a gap was still observed between the mid-portion of the array and the modiolus (the first white marker was seen in the RW). When we inserted the electrode more deeply, the mid-portion of the array was pushed away from the modiolus (the second and third white markers were seen in the RW). After applying the pull-back technique, the gap observed during the initial insertion disappeared, resulting in an optimal perimodiolar position (the first white marker was once again visible in the RW).

**Conclusion:**

This temporal bone study demonstrated that when applying the pull-back technique for the 532 slim modiolar electrode, a closer proximity to the modiolus was achieved when the first white marker of the electrode array was visible in the round window.

## 1. Introduction

Cochlear implantation is the treatment of choice for severe to profound hearing loss. Since the first nonexperimental cochlear implantations, there have been more than 500,000 implantations worldwide. In recent years, the design of cochlear implant arrays has changed [1]. One notable innovation was the introduction of perimodiolar electrodes in the late 1990s. The electrode in a cochlear implant system is the central factor for hearing performance, as it is the interface between the device and the auditory pathway of the recipient [2]. Current commercially available electrode arrays can be divided into two classes: straight lateral wall electrode arrays and precurved perimodiolar or midmodiolar electrode arrays [3]. The distance between the modiolus and the electrode array has become the focus of many discussions and studies for a variety of reasons.

Positioning the electrodes closer to the spiral ganglion with the goal of reducing the current spread during electrical stimulation has been shown to improve hearing outcomes [1]. An electrophysiological effect was demonstrated for the first time by Shepherd, who reported reduced electric auditory brainstem response thresholds while positioning the electrode array closer to the modiolus [4]. Further studies have proven that the perimodiolar electrode position decreases channel interactions and neural response telemetry thresholds and leads to better speech understanding [5, 6]. Some other benefits, like a decrease power consumption and an increase in the dynamic range, have also been reported [5, 7]. The comfort level among users implanted with a perimodiolar electrode array seems to be higher than among users implanted with a straight lateral wall array [8]. Holden et al. concluded that total insertion depth was not associated with better speech discrimination outcomes; however, the distance from the electrodes to the modiolus did indicate a significant influence [9]. Any surgery technique or strategy that prompts a better perimodiolar positioning of the electrode array could therefore lead to an increase in hearing outcomes.

The perimodiolar electrode arrays can be complemented with a surgical manoeuvre called the pull-back technique. This technique consists of a normal insertion of the electrode with a subsequent pulling back until the first white marker of the electrode array becomes microscopically visible in the round window (RW). A better perimodiolar position of the electrode is achieved by this manoeuvre. The pull-back technique is assumed to bear no serious risks to the cochlear microstructures and has shown to be reliable and reproducible [10]. Electrophysiological changes have also been reported in different studies applying this intervention. In one study, the spread of excitation decreased significantly in the medial and basal part of the cochlea after the pull-back technique was applied [11]. Another study demonstrated that electrically evoked action potential amplitudes at a fixed stimulus level increased after implementing this procedure [12]. The array inside the scala tympani is invisible to the surgeon, so the proximity of the electrode array to the modiolar wall is generally unknown [1]. The variable amount of pull-back and the variability of sizes of the human cochlea make this procedure different for each electrode array [13]. Clear surgical guidelines have been published for the Nucleus Advance electrode and the Advanced Bionics Helix electrode [14, 15]. This study focuses its attention on the recently developed 532 slim modiolar electrode.

The 532 slim modiolar electrode allows closer placement to the modiolus due to a higher degree of precurvation in comparison to the Nucleus Contour Advance Electrode [16]. Therefore, the goal of this work is to investigate the intracochlear movements and pull-back technique for the 532 slim modiolar electrode.

## 2. Material and Methods

A decapping procedure of the cochlea was performed on 5 randomly chosen human formaldehyde treated temporal bones (3 left and 2 right). This consisted of removing the roof of the scala vestibuli until a full visual assessment of the basilar membrane was possible. The basilar membrane was also removed to obtain a panoramic view of the intrascalar position of the array in the scala tympani. A RW approach was performed for the insertion of the electrode array. This procedure was made under moisturized conditions and microscopic control to simulate an authentic situation. The sizes of the 5 temporal bones/cochleas (distance from the RW to the furthest part from the lateral wall of the cochlea, Distance A) were measured. Because human cochleae vary in size, the 532 slim modiolar electrode has three white markers for insertion. The marker closest to the RW is called number 1, the following number 2, and the final marker number 3. The electrode array was inserted with the recommended sheath insertion and removal technique. The intracochlear movements were microscopically examined and digitally captured (Zeiss OPMI Pentero 900). Three situations for the 5 temporal bones were analysed and digitally captured: initial insertion (to the first marker), overinsertion (up to the third marker), and the pull-pack position (to the first marker). The position of the three white markers of the electrode array in relation to the RW was also evaluated when performing these three actions. The change in distance between the center of the modiolus and the contact 11 was measured based on the digital capturing (Table 1).

## 3. Results

**Figure 1:**
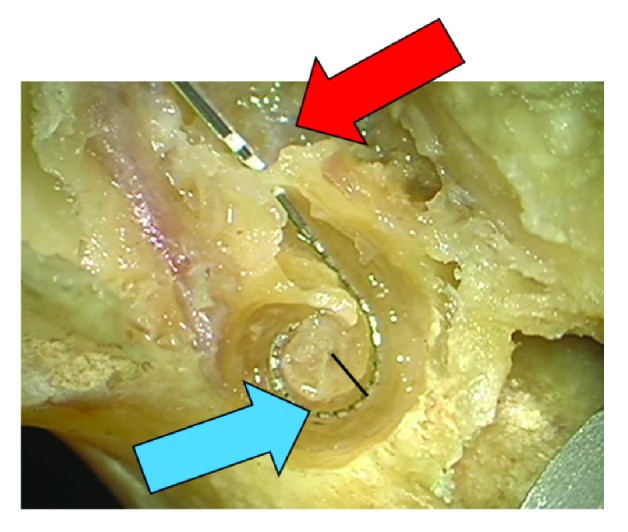
Initial insertion of the electrode up to the first marker (red arrow) with distance of the electrode at the modiolus (blue arrow). Black line indicates distance between modiolus and contact 11.

**Figure 2:**
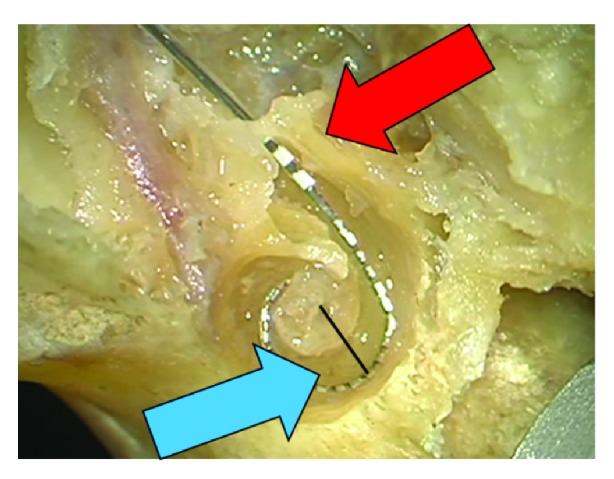
Overinsertion of the electrode up to the third marker (red arrow) with distance of the electrode at the modiolus (blue arrow). Black line indicates distance between modiolus and contact 11.

**Figure 3:**
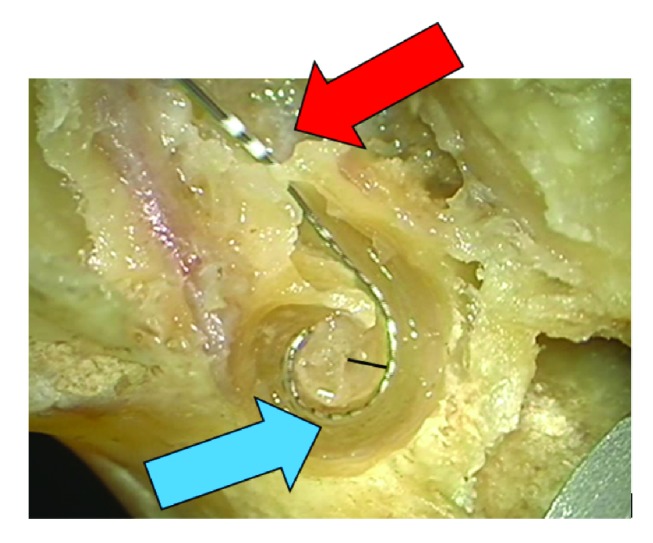
Pull back of the electrode up to the first marker (red arrow) with close position of the electrode at the modiolus (blue arrow). Black line indicates distance between modiolus and contact 11.

The mean size of the temporal bones/cochleas was 8.64mm, the longest Distance A was 9.5mm, and the shortest was 8 mm. For all 5 temporal bones, the same pattern was observed when analysing the three situations, as previously described. The initial insertion achieved an acceptable perimodiolar position of the electrode array, but a gap was still observed between the mid-portion of the array and the modiolus (Figure 1). In this scenario, only the first white marker was observed in the RW. When the array was inserted more deeply, the mid-portion of the array was pushed away from the modiolus, resulting in an unfavourable perimodiolar position (Figure 2). In this case, the third white marker was seen in the RW. Finally, the pull-pack technique was applied, resulting in an optimal perimodiolar position of the electrode array. When applying this technique, we found that the gap observed during the initial insertion disappeared and that the first white marker of the electrode array was already visible in the RW. No tip movements were detected during this procedure. The same situation repeated itself while applying this technique to the 5 different temporal bones. We did not find a correlation between the size or side (left or right temporal bone) of the cochlea and the amount of pull-back applied. The closest perimodiolar position for all 5 temporal bones was attained when the first white marker of the electrode array was visible in the RW after applying the pull-back technique (Figure 3). Measurement showed a mean initial distance between modiolus center and contact 11 of 1,9 mm (SD 0,2mm). Overinsertion resulted in a distance of 2,5 mm (SD 0,4 mm). The pull-back finally resulted in a distance of 1,5 mm (SD 0,2 mm).

## 4. Discussion

Since its introduction, each component of the cochlear implant has been the subject of continual research and innovation to achieve the best performance in speech perception and production. In particular, special attention has been paid to electrode placement and design. Another important feature is atraumatic insertion. To limit trauma during electrode insertion, the array should be positioned entirely within the scala tympani [17]. Advantages and disadvantages have been described for each type of electrode array. One disadvantage of the straight electrodes is their final position, as they lay at the lateral wall of the cochlea, which is far away from the neural elements in the modiolar area. By contrast, preformed electrode arrays are fabricated in a spiral configuration and adjusted to the human cochlea's modiolar area. These electrode arrays were designed for intracochlear placement next to the modiolus [18]. This position leads to a narrower electric stimulation, a lower current spread to the adjacent neural population, a lower channel interaction, and a reduced risk of facial nerve stimulation. As a consequence, the behavioural and electrically evoked compound action potential thresholds are reduced with a wider dynamic range [17]. However, these perimodiolar designs also have disadvantages, as these electrodes, until recent redesigns, have had a larger diameter and were associated with a higher risk of insertion trauma. Another problem with perimodiolar electrodes until now is that, with these precurved arrays, dislocation occurs in up to 26% of the cases, which is associated with poorer outcomes [16]. Additionally tip foldovers can occur [19]. With straight flexible electrode arrays, the incidence of dislocation has been found to be lower [18].

The development of an electrode array that lies close to the modiolus, which can be inserted with minimal trauma to the delicate cochlear structures and which stays within the scala tympani, is technically challenging [8]. In an aim to achieve these objectives, a thin, precurved electrode was recently developed by Cochlear Ltd. and approved for clinical use in 2016 [17, 20]. The CI532 is held straight prior to insertion by an external polymer sheath, which is removed after full insertion of the array. This new kind of electrode is even closer to the modiolus. The elimination of the internal stylet and surrounding silicone rubber reduces the electrode volume by up to 75%, resulting in dimensions equivalent to that of the current lateral wall electrodes [17]. In comparison to the Nucleus Contour Advance, the new CI532 has a diameter of 0.5 mm at the position of the most basal electrode, reducing to 0.4 mm at the apex (the corresponding dimensions of the Nucleus Contour Advance are 0.8 mm and 0.5 mm). This gives the CI532 a cross-sectional area about 40% that of the Nucleus Contour Advance [16]. Potential advantages of this new design include minimal insertion trauma and consistent perimodiolar location within the scala tympani [20]. A study by Aschendorff et al. found that the CI532 achieved the design goal of producing no trauma, as indicated by 100% scala tympani placement, while achieving consistent and close modiolar proximity [16]. However, a study by McJunkin et al. reported that 13% of the CI532 implants dislocated into the scala vestibule [20]. These results for the CI532 are lower than those reported for the previous Nucleus Contour Advance.

An intraoperative intervention regarding the position of perimodiolar electrodes can further reduce the distance of the electrode contacts to the modiolus [11]. This intervention, called the pull-back technique, has shown favourable results for the Nucleus Contour Advance and the Advanced Bionics Helix electrode [14]. The pull-back technique seems to cause a better perimodiolar position of the electrode arrays [12]. Basta et al. proved that the spread of excitation was significantly reduced at basal, middle, and apical electrodes in the electrode pull-back group for the Nuclear Contour Advance, while a significantly smaller frequency difference limen was observed with the 4 kHz. This means that the pull-back technique has its greatest effect in the basal region of the cochlea [11]. Another study using the Advanced Bionics Helix electrode showed similar results after applying the pull-back technique. The spread of excitation showed a significant decrease of the intracochlear field in all three contacts (basal, middle, and apical). The recommended pull-back distance for the Advanced Bionics Helix electrode was about 1 mm [15]. As the pull-back technique can be performed in different ways (insertion depth, amount of pull-back, and variability of the human cochlea), surgical guidelines are required [14]. Therefore, it was the goal of the present study to estimate the change in position of the CI532 while being pulled back in a series of temporal bones. The best pull-back distance is defined as the pull-back distance while the tip is still in its unchanged apical position and the middle part of the electrode is maximally approximated to the modiolus [15]. This situation was observed and digitally captured in all of the temporal bones where we performed the procedure. In our study, we did not see any tip foldover. This was not the case in another study where tip foldovers occurred in 1% to 8% of the CI532 implants, which is noteworthy [20, 21]. Direct measures of electrode to modiolus distance, even from the best quality CT imaging available, are problematic due to residual electrode artefacts blurring the boundary between the medial and medial wall [16]. This is why cadaveric studies like ours provide the best way to assess the electrode to modiolus distance. The study by Aschendorff et al. came to similar conclusions as ours. In their study, the electrode to modiolus distance was evaluated using CT. As in our study, advancing the CI532 electrode array past the first white marker position into the cochlea opening was undesirable, as it does not result in greater total insertion depths and only serves to increase the insertion depth of the first electrode contact and move basal electrodes away from the modiolus [16]. Ramos-Macías et al. also evaluated electrode to modiolus distance using CT and demonstrated that it was constant in all electrode arrays and less than 0.3 mm [18]. Unfortunately, the pull-back technique was not performed in either of the studies.

The CI532 combines important electrode characteristics: a position closest to the modiolus, limited insertion trauma, and positioning within the scala tympani [17]. Adding a surgical technique modification called the pulled-back to this array could be of promising interest in frequency discrimination and number of virtual channels [11, 15].

## 5. Conclusion

This temporal bone study demonstrated that when applying the pull-back technique for the 532 slim modiolar electrode, a closer proximity to the modiolus was achieved when the first white marker of the electrode array was visible in the RW.

## Figures and Tables

**Table 1:** Temporal bone specific effect of insertion, over insertion and pull back on distance between modiolus center and contact 11.

in mm	*TB1*	*TB 2*	*TB 3*	*TB 4*	*TB 5*
*Initial Insertion*	2	1,9	2	1,5	2,1
*Over Insertion*	3,1	2,5	2,4	2,1	2,4
*Pull back*	1,4	1,5	1,5	1,2	1,8

## Data Availability

The data used to support the findings of this study are available from the corresponding author upon request.
